# ROS-Mediated NLRP3 Inflammasome Activity Is Essential for Burn-Induced Acute Lung Injury

**DOI:** 10.1155/2015/720457

**Published:** 2015-10-20

**Authors:** Shichao Han, Weixia Cai, Xuekang Yang, Yanhui Jia, Zhao Zheng, Hongtao Wang, Jun Li, Yan Li, Jianxin Gao, Lei Fan, Dahai Hu

**Affiliations:** Department of Burns and Cutaneous Surgery, Xijing Hospital, Fourth Military Medical University, Xi'an, Shaanxi 710032, China

## Abstract

The NLRP3 inflammasome is necessary for initiating acute sterile inflammation. However, its role in the pathogenesis of burn-induced acute lung injury (ALI) is unknown. This study aimed to determine the role of the NLRP3 inflammasome and the signaling pathways involved in burn-induced ALI. We observed that the rat lungs exhibited enhanced inflammasome activity after burn, as evidenced by increased levels of NLRP3 expression and Caspase-1 activity and augmented inflammatory cytokines. Inhibition of NLRP3 inflammasome by BAY11-7082 attenuated burn-induced ALI, as demonstrated by the concomitant remission of histopathologic changes and the reduction of myeloperoxidase** (**MPO) activity, inflammatory cytokines in rat lung tissue, and protein concentrations in the bronchoalveolar lavage fluid (BALF). In the *in vitro* experiments, we used AMs (alveolar macrophages) challenged with burn serum to mimic the postburn microenvironment and noted that the serum significantly upregulated NLRP3 inflammasome signaling and reactive oxygen species (ROS) production. The use of ROS scavenger N-acetylcysteine (NAC) partially reversed NLRP3 inflammasome activity in cells exposed to burn serum. These results indicate that the NLRP3 inflammasome plays an essential role in burn-induced ALI and that burn-induced NLRP3 inflammasome activity is a partly ROS-dependent process. Targeting this axis may represent a promising therapeutic strategy for the treatment of burn-induced ALI.

## 1. Introduction

Acute lung injury (ALI) following severe burns remains a prominent source of morbidity and mortality among critically ill patients. Severe burn injury, consisting of several damage factors like trauma stress, thermal attack, and tissue hypoperfusion, triggers an abundance of detrimental secondary effects, including inflammation, oxidative stress, and apoptosis, which results in either ALI or acute respiratory distress syndrome (ARDS) [1–3]. The primary pathological mechanisms underlying ALI are vascular endothelial and alveolar epithelial cell damage, which result in the destruction of blood-alveolar barrier. This destruction yields pulmonary edema, intrapulmonary hemorrhage, and severely impaired gas exchange [4]. Although the precise mechanisms underlying the development of ALI following severe burn injury remain unclear, inflammatory response accounts for major reasons. Literatures suggest that inflammatory responses following burn insult are associated with robust release of proinflammatory cytokines and activation of sympathetic inflammatory signaling pathways [5, 6].

Macrophages play a central role in both the maintenance of immunological homeostasis and host defense. In response to inflammatory stimuli, macrophages are the primary source of cytokines in lungs [7] and play an important role in the pathogenesis of lung injury, initiating inflammatory responses and promoting neutrophil infiltration and tissue damage in the lungs [8]. Macrophages can be rapidly activated by burn injury and may therefore play an important role in burn-induced ALI [9]. Macrophage depletion improves alveolar barrier dysfunction and lung inflammatory response caused by severe burns [10].

Increasing amounts of evidence have indicated that cytokines, such as interleukin-1 beta (IL-1*β*) and interleukin-18 (IL-18), participate in inflammation and innate immunity response [11, 12]. IL-1*β* is one of the most potent inflammatory initiating cytokines observed in patients suffering from ALI and induces the production of additional cytokines [13]. IL-18 also functioned as a proinflammatory cytokine in a model of acute lung inflammation, serving as both an autocrine activator and a facilitator of additional inflammatory mediators [14]. The antibody (Ab) neutralization of either IL-1*β* or IL-18 has been found to attenuate ALI in different rodent models [7, 14]. Elevated expression of both IL-1*β* and IL-18 following burn injury has also been verified [1, 15, 16]. Given the prominent roles played by inflammatory initiating cytokines in systemic liberation of inflammatory mediators and augmentation of inflammatory response, this study was undertaken to determine whether the control of their release attenuates burn-induced ALI.

IL-1*β* and IL-18 are primarily produced by inflammasomes, intracellular macromolecular complexes that function as a platform for Caspase activation and trigger subsequent proteolytic maturation of proinflammatory cytokines, such as IL-1*β* or IL-18. Most inflammasomes contain an NLR family protein, an apoptosis-associated speck-like protein that contains a Caspase-recruitment domain (ASC), and a cysteine protease Caspase-1. Several types of inflammasome complexes have been described in literature, each of which has been named based on the specific NLR it contains. The NLRs that participate in the inflammasome complexes include NLRP1, NLRP3, NLRP6, NLRP7, NLRP12, NLRC4, AIM2, and IFI16 [17]. Of them, the NLRP3 inflammasome is the most studied and important member of inflammasome complex family, which has been demonstrated to associate with ALI. NLRP3 inflammasomes are expressed in many types of immune cells, including monocytes, macrophages [18], and T cells [19], and nonimmune cells, such as fibroblasts, myofibroblasts [20], keratinocytes [21], endothelia cells [22], and hepatic stellate cells [23]. Substantial evidence has indicated that the NLRP3 inflammasome initiates innate immunity and augments inflammatory responses. Previous studies had demonstrated that the development of hemorrhagic shock-induced ALI required the NLRP3 inflammasome and that the inhibition of NLRP3 attenuated hemorrhagic shock-induced ALI [24, 25]. Therefore, we speculated that the NLRP3 inflammasome may be involved in burn-induced ALI.

The NLRP3 inflammasome can be activated by many factors, including environmental irritants, endogenous danger signals, pathogens, and various pathogen-associated molecular patterns (PAMPs). Moreover, multiple cellular signaling mechanisms are involved in NLRP3 activation, including potassium efflux, lysosome damage, and mitochondrial damage [26]. Moreover, reactive oxygen species (ROS) have been identified as an important NLRP3 inflammasome activator in settings of various diseases, such as hepatic ischemia/reperfusion injury [27], hemorrhagic shock-induced ALI [25], and hypertension-induced cardiac remodeling [28]. However, the role of ROS in activating the NLRP3 inflammasome in the setting of burn-induced ALI is unclear.

Therefore, we investigated the expression of the NLRP3 inflammasome in a rat burn model and determined the biological functions of NLRP3. We also investigated the signaling pathways that trigger activation of the NLRP3 inflammasome in the setting of burn-induced ALI.

## 2. Materials and Methods

### 2.1. Animal Model and Experimental Groups

Healthy adult male Sprague-Dawley rats (200–250 g) were used for the experiments. Rats were randomly divided into the following three groups: a sham group (*n* = 6), a burn group (*n* = 24), and a burn + BAY11-7082 group (*n* = 12). Rats were anesthetized (1% pentobarbital sodium 5 mL/kg) 24 h before burn experiment, and their dorsal fur was clipped using an electric clipper. Animals were subsequently placed into a template constructed to estimate 30% of their total body surface area (TBSA) and subjected to full-thickness burn injury via exposure to water maintained at 92°C for 18 seconds [29, 30]. Rats subsequently received an intraperitoneal injection of 5 mL normal saline for resuscitation. The sham burn control rats were subjected to an identical procedure; however, they were immersed in room temperature water, and no fluid resuscitation was administered. Six rats were sacrificed at 6, 12, 24, and 48 hours, respectively, following burn injury, and their tissues were sampled.

BAY11-7082 has previously been used to block the NLRP3 inflammasome [22]. Therefore, NLRP3 inflammasome blocker BAY11-7082 (Selleckchem, USA) was administered to burn + BAY11-7082 group immediately after burn injury in order to study the effect of the NLRP3 inflammasome. Rats in burn + BAY11-7082 group were intraperitoneally injected with 15 *μ*mol/kg BAY11-7082 in 1 mL/kg DMSO, whereas rats in sham group and burn group were treated with vehicle control (1 mL/kg DMSO). Rats were sacrificed and their tissues were sampled 24 and 48 hours following burn injury. Each tissue sample was divided into three parts for Western blotting, quantitative real-time polymerase chain reaction (PCR), and histological examination.

The rats were purchased from the Experimental Animal Center of the Fourth Military Medical University and housed under specific pathogen-free conditions. The animal experiments were approved by the Experimental Animal Committee of the Fourth Military Medical University.

### 2.2. Sample Collection

Animals were sacrificed under anesthesia at each of the aforementioned time points following burn injury. Blood samples were taken from aorta ventralis centrifuged at 1500 g for 10 minutes; serum was collected and stored at −80°C until needed. Animals' sternums were cut open at the midline, and trachea and lungs were exposed. Bronchoalveolar lavage fluid (BALF) was obtained by cannulating the trachea with an infusion tube. An infusion tube was inserted into trachea and ligated by a thread. Four milliliters of cold phosphate-buffered saline was injected into the lung using a syringe fitted with the infusion tube. The phosphate-buffered saline was allowed to remain in the lung for 30 sec before being removed with a syringe, a process that was repeated three times with the same solution. The BALF samples were centrifuged at 1500 g for 10 minutes, and the supernatant was collected and stored at −80°C until needed. The lower lobe of the right lung was ligated prior to the bronchoalveolar lavage before being excised and divided into three parts for real-time PCR, Western blotting, and histopathological analysis.

### 2.3. Enzyme-Linked Immunosorbent Assay (ELISA) for IL-1*β* and IL-18 Levels Determination of Serums and Cell Supernatants

The levels of IL-1*β* and IL-18 in serums or cell supernatants were quantified using ELISA kits (Jiancheng, Nanjing, China), according to the manufacturers' protocols. An ELX808 microplate reader was used to measure the optical density (OD) values at a wavelength of 450 nm.

### 2.4. Histological Evaluation

The lung tissue samples were obtained from the different groups at the indicated time points. Tissues were fixed in 4% paraformaldehyde and embedded in paraffin blocks using an automated processor. Sections were cut at a thickness of 4 *μ*m and mounted onto slides. Some sections were stained with hematoxylin and eosin (H&E), whereas others were prepared for immunohistochemistry using standard methods. Two blinded examiners evaluated lung injury using the scoring standard described below. Intra-alveolar edema, intra-alveolar hemorrhage, and neutrophil infiltration in the tissues were scored on a scale from 0 to 4 (0, absent; 1, mild; 2, moderate; 3, severe; 4, overwhelming). The total histology score is expressed as the sum of the scores for each parameter as described previously [31]. A rat anti-NLRP3 polyclonal antibody (Biorbyt, Cambridge, UK) was used at a 1 : 200 dilution to immunostain tissues for NLRP3. The NLRP3 protein was visualized using diaminobenzidine. Images were obtained using an Olympus IX71 light microscope (Olympus, Tokyo, Japan) with the QImaging software (Surrey, BC, Canada). The expression of NLRP3 was quantitatively assessed by counting the numbers of positive cells in five different fields (mm^2^) per lung.

### 2.5. Total Protein Assay in the BALF

The BALF protein concentrations were measured using a bicinchoninic acid protein assay kit (Pierce, Rockford, IL) and a DU-800 spectrophotometer (Beckman, Fullerton, CA) at a wavelength of 562 nm.

### 2.6. Measurement of Lung Myeloperoxidase (MPO) Activity

The MPO activity was spectrophotometrically assayed using appropriate kits (Jiancheng, Nanjing, China). One hundred milligrams of lung tissue was homogenized and fluidized in extraction buffer to obtain a 5% homogenate. The sample, including 0.9 mL of homogenate and 0.1 mL of reaction buffer, was heated to 37°C in a water bath for 15 minutes, and the enzymatic activity was subsequently measured using a DU-800 spectrophotometer (Beckman, Fullerton, CA) at a wavelength of 460 nm. This activity is expressed as units per gram of weight.

### 2.7. Isolation of AMs

AMs were isolated by bronchoalveolar lavage (BAL) as described previously [17]. For AMs isolation, rats were anesthetized and sacrificed by aorta ventralis exsanguination. The method of BALF collection was described in the former text. BALF was centrifuged at 1500 rmp for 15 min at 4°C. The cells were then incubated in 100 mm culture dishes for 90 min at 37°C. Cells adhering to the bottom of dish were collected and replated for further experimental use.

### 2.8. Cell Culture

Cell cultures were maintained at 37°C in humidified incubators that contained an atmosphere of 95% air and 5% CO_2_. AMs (alveolar macrophages) were cultured in collagen I-coated 35 mm^2^ plates and maintained in RPMI1640 medium supplemented with 10% fetal bovine serum (Gibco), 2 mM glutamine, and 1% streptomycin/penicillin. AMs were plated at a density of 2 × 10^5^ cells/plate; 2 hours before cell experiment, the macrophages were starved without fetal bovine serum. To challenge AMs, the cells were incubated with either sham serum (SS) or burn serum (BS) (10% vol/vol) for either 24 or 48 hours, and some of cells were treated with 10 *μ*M BAY11-7082 immediately after burn serum or sham serum was added to the culture. In the remaining experiments, cells were challenged with burn serum, in the presence of ROS scavenger, 1 *μ*M N-acetylcysteine (NAC) (Sigma-Aldrich, St. Louis, MO).

### 2.9. Flow Cytometry Analysis

AMs were subjected to intracellular ROS staining using a Reactive Oxygen Species Assay Kit (Beyotime, Shanghai, China). Briefly, cells were incubated with 5 *μ*M DCFH-DA for 30 min at 37°C. Cells were washed twice with PBS and harvested for fluorescence detection using a FACS Calibur (BD, Franklin Lakes, NJ). In some experiments, Rosup was used as a positive control. The relative levels of ROS were quantitatively determined based on the mean fluorescence intensity (MFI).

### 2.10. Real-Time Reverse Transcription PCR (qRT-PCR)

The total mRNA expression levels of NLRP3, Caspase-1, IL-1*β*, and IL-18 were analyzed via PCR; total RNA was extracted and purified using TRIzol (Invitrogen, CA, U.S.) and a Total RNA Isolation Kit (Takara, Otsu, Shiga, Japan). The purity of RNA was subsequently determined based on A260/A280 ratio. The mRNA was reverse-transcribed into cDNA using a Prime Script RT Reagent Kit (Takara) in accordance with manufacturer's instructions. Using SYBR Premix Ex Taq Kit (Takara, Japan) and specific primers, the expression levels of target gene mRNAs were measured with a Bio-Rad IQ5 Real-Time System (Bio-Rad, Hercules, CA). GAPDH was used as a housekeeping gene for mRNA analysis. The primer sequences are listed in Table 1.

The following thermal cycle condition was employed to reverse-transcribe the mRNA: an initial denaturation at 95°C for 30 sec, followed by 40 cycles of denaturation at 95°C for 15 sec, annealing at 60°C for 30 sec, and elongation at 72°C for 10 sec. Relative concentrations of target genes were determined based on the cycle threshold (Ct). The qRT-PCR data were exported and processed using the ΔΔCt method.

### 2.11. Western Blot Analysis

Thirty micrograms of total protein was loaded per well, electrophoresed on a 12% sodium dodecyl sulfate polyacrylamide gel, and transferred onto a polyvinylidene difluoride membrane. The membrane was subsequently probed with polyclonal rabbit anti-rat NLRP3 (1 : 400; Biorbyt, Cambridge, UK) and Caspase-1 antibodies (1 : 200; Santa Cruz, USA) for 12 hours at 4°C. Membranes were subsequently incubated with appropriate HRP-conjugated secondary antibody at room temperature for 1 h. Immunoreactive traces were detected using a chemiluminescent HRP Substrate ECL Kit (Millipore, USA). The density of each protein band on the membrane was scanned using a FluorChem FC system (Alpha Innotech, San Jose, California, USA) and is presented as a densitometric ratio between the protein of interest and the loading control.

### 2.12. Statistical Analysis

Histopathological scores are expressed as means ± SEMs. Intergroup differences in histopathological scores were tested using the nonparametric Kruskal-Wallis method, followed by Nemenyi test for multiple comparisons. Measurement values are expressed as means ± SEMs. The significance of differences was assessed with an analysis of variance (ANOVA) followed by Bonferroni* t*-test using the SPSS 17.0 software (Chicago, IL, USA). *P* < 0.05 was considered to indicate significant differences.

## 3. Results

### 3.1. Burn Injury Increased the Levels of IL-1*β* and IL-18 in Rat Serum and Lung Tissue

**Figure 1:**
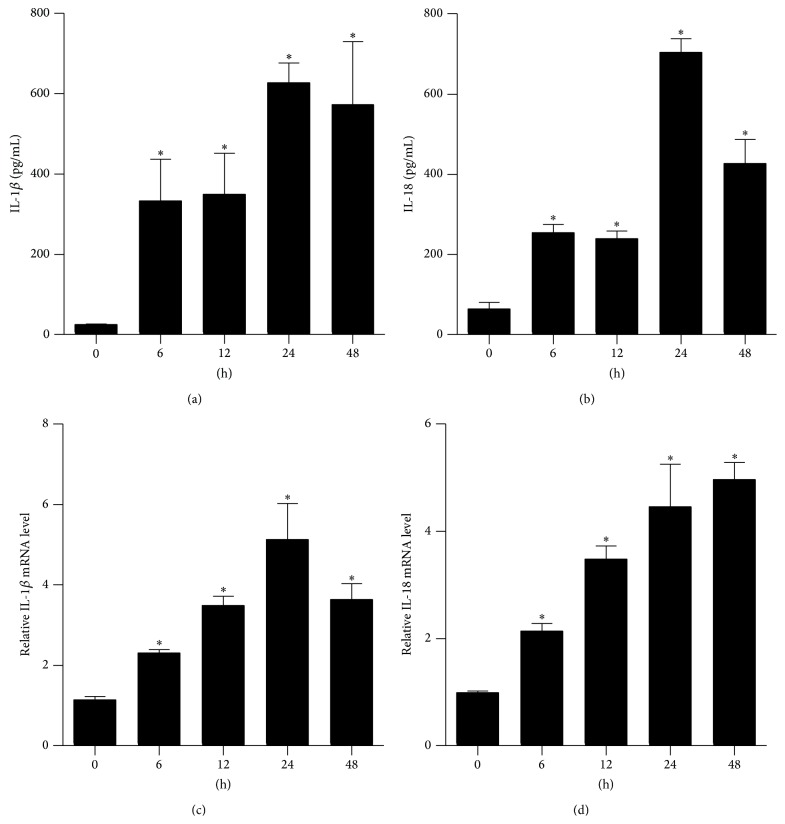
The expression of IL-1*β* and IL-18 in serum and lung tissue from severely burned rats over time. Rats were subjected to burn injury. IL-1*β* (a) and IL-18 (b) in serum were determined using ELISA. The mRNA levels of IL-1*β* (c) and IL-18 (d) in rat lungs were analyzed by real-time PCR and normalized against GAPDH mRNA level. The values presented are mean ± SEM (*n* = 6 per group). ^*∗*^ *P* < 0.05, compared to the value at 0 h.

Severe burn injury triggers multiple inflammatory reactions. IL-1*β* and IL-18 act as inflammatory initiating cytokines, propagating injury signals and triggering an inflammatory cascade. Therefore, we analyzed the production of IL-1*β* and IL-18 in rat serum and lung tissue samples. The ELISA and qRT-PCR results demonstrated that the levels of IL-1*β* (Figures 1(a) and 1(c)) and IL-18 (Figures 1(b) and 1(d)) were significantly increased compared with the sham group (0 h) over time; the expression levels of IL-1*β* and IL-18 peaked at either 24 h or 48 h.

### 3.2. Burn Injury Resulted in Elevated NLRP3 and Caspase-1 mRNA and Protein Expression Levels in Rat Lungs

**Figure 2:**
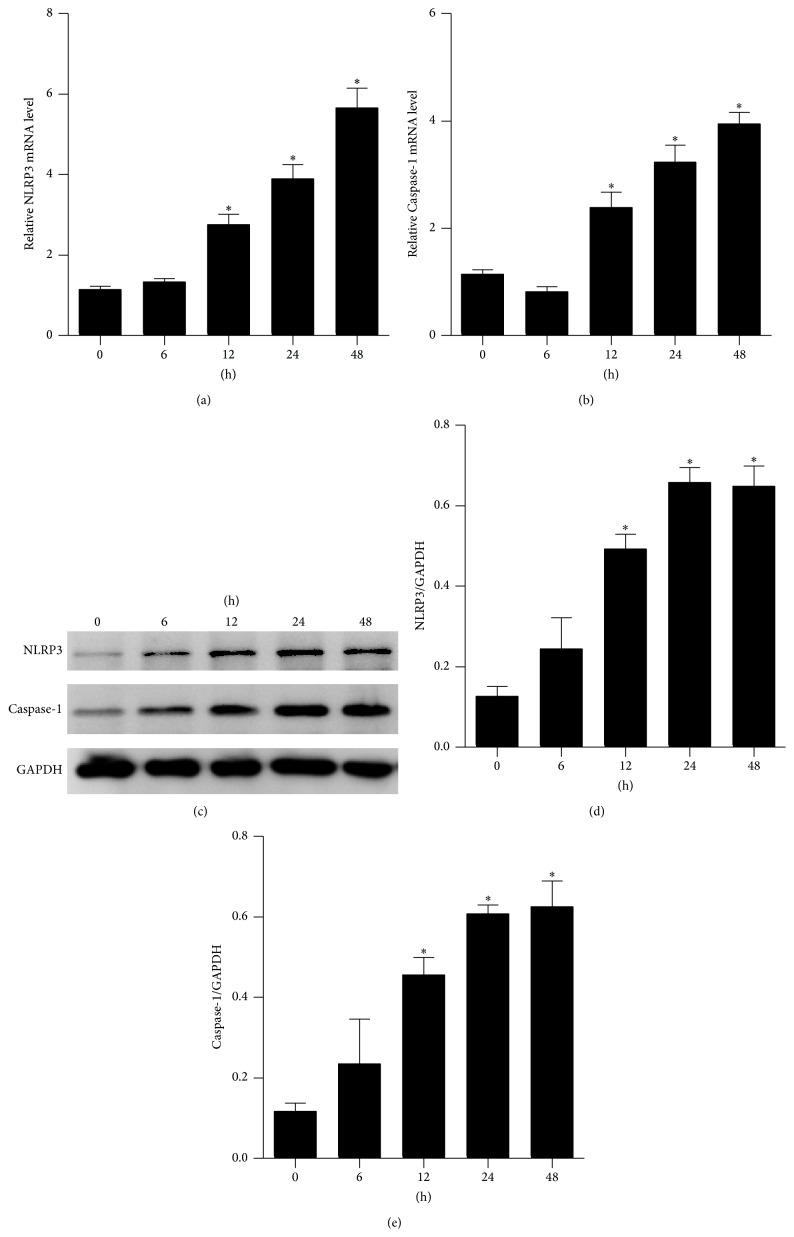
NLRP3 and Caspase-1 were activated in rat lungs after burn. Rats were subjected to burn injury. The mRNA expression ((a) and (b)) of NLRP3 and Caspase-1 in lung tissue after burn over time. The protein levels ((c), (d), and (e)) of NLRP3 and Caspase-1 in lung tissue from severely burned rats over time. The values presented are mean ± SEM (*n* = 6 per group). ^*∗*^ *P* < 0.05, compared to the value at 0 h.

The NLRP3 inflammasome activates Caspase-1, which results in the maturation of pro-IL-1*β* and pro-IL-18. To determine whether NLRP3 and Caspase-1 are activated in lungs following burn injury, we assessed the expression of NLRP3 and Caspase-1 at both mRNA and protein levels by qRT-PCR and Western blotting, respectively. Results indicated that the NLRP3 and Caspase-1 mRNA levels did not differ between the sham and burned rats at 6 h but significantly increased at 12 h and remained elevated until 48 h after burn (Figures 2(a) and 2(b)). The NLRP3 and Caspase-1 protein levels corresponded to their mRNA expression levels (Figures 2(c), 2(d), and 2(e)). These results indicated that the NLRP3 inflammasome may be involved in burn-induced ALI.

### 3.3. The NLRP3 Inflammasome Inhibitor BAY11-7082 Attenuated Burn-Induced NLRP3 Activity in Lungs

**Figure 3:**
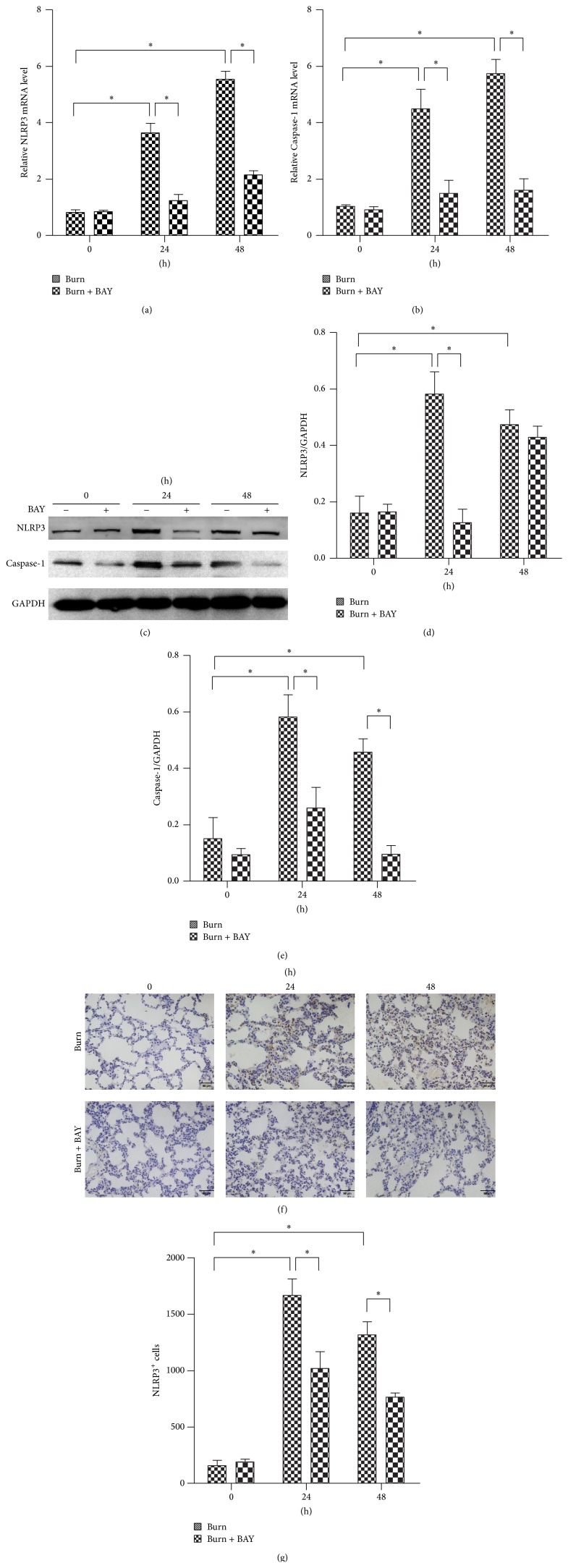
NLRP3 inflammasome inhibitor BAY11-7082 attenuated burn-induced NLRP3 activation in lungs. Rats were subjected to burn injury. BAY11-7082 was administered intraperitoneally immediately after burn injury in rats. At 24 h and 48 h after burn injury, lung tissue was collected. The mRNA expressions ((a) and (b)) and protein level change ((c), (d), and (e)) of NLRP3 and Caspase-1 were analyzed by real-time PCR and immunoblots in lung tissue after burn with BAY11-7082 treatment. The NLRP3 positive signal was determined by immunohistochemistry (f) and was quantified (g) in (f) per mm^2^ in lung tissue after burn with BAY11-7082 treatment. The values presented are mean ± SEM (*n* = 6 per group). ^*∗*^ *P* < 0.05.

BAY11-7082 is an NLRP3 inflammasome inhibitor. To determine whether BAY11-7082 successfully decreased the NLRP3 activity, the expressions of NLRP3 and Caspase-1 at both the mRNA and protein levels were measured by qRT-PCR and Western blotting, respectively. In the previous experiment, we concluded that the NLRP3 inflammasome was significantly activated at 24 h or 48 h following burn injury; therefore, we selected the time points in question for further study.  Figure 3 demonstrates that treatment with BAY11-7082 significantly inhibited NLRP3 inflammasome signaling, as evidenced by 2-3-fold decreases in NLRP3 and Caspase-1 mRNA and protein expression. The inhibition of NLRP3 inflammasome signaling was further confirmed via immunohistochemistry, as depicted in Figures 3(f) and 3(g).

### 3.4. BAY11-7082 Alleviated Burn-Induced ALI

**Figure 4:**
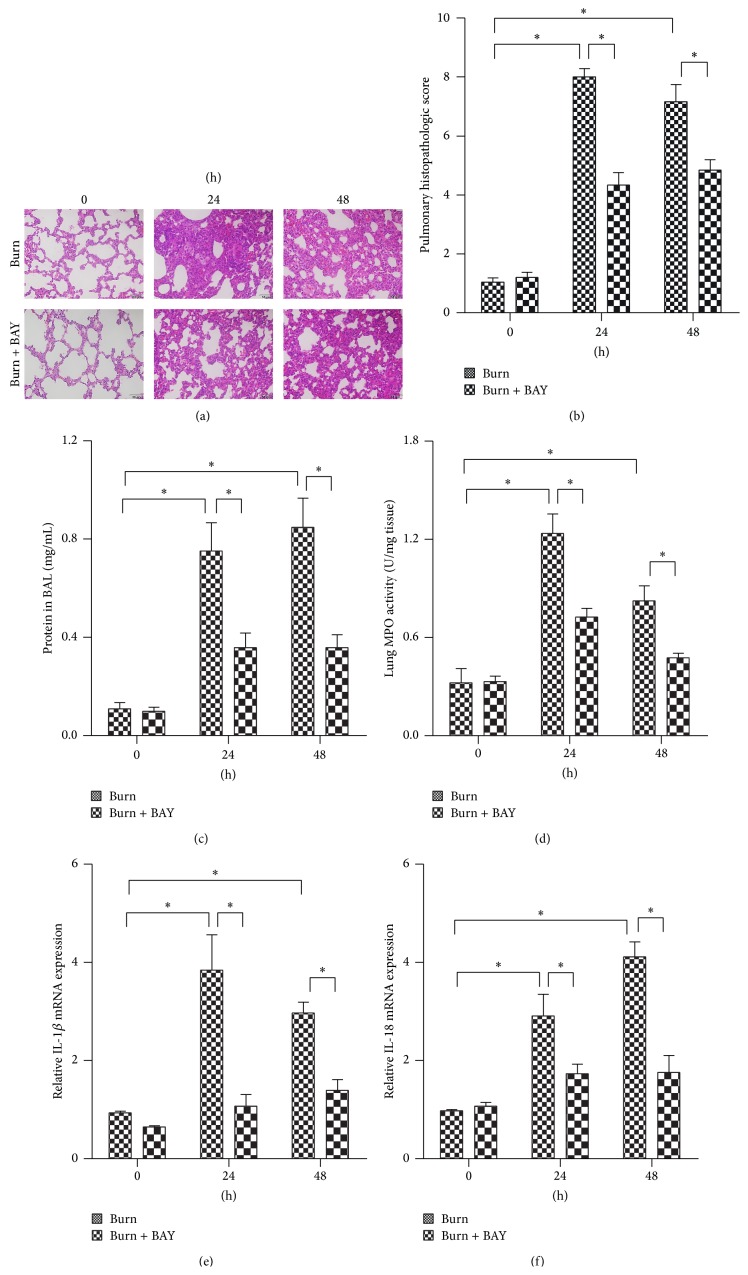
BAY11-7082 alleviated the burn-induced ALI. Rats were subjected to burn injury. BAY11-7082 was administered intraperitoneally immediately after burn injury in rats. At 24 h and 48 h after burn injury, lung tissue was collected. Histopathological changes (a) were assessed by H&E staining and the pulmonary histologic score (b) was counted. Total protein contents in BALF (c) were examined by BCA protein assay kit. The levels of MPO in lung homogenate (d) were tested by MPO kits. IL-1*β* (e) and IL-18 (f) mRNA expression in rat lungs were analyzed by real-time PCR. The values presented are mean ± SEM (*n* = 6 per group). ^*∗*^ *P* < 0.05.

Pretreatment with BAY11-7082 protected against burn-induced ALI. As demonstrated in burn group of Figure 4(a), the lungs exhibited significant histopathologic changes, including alveolar wall thickening, neutrophil infiltration, pulmonary edema, and alveolar hemorrhage. However, pretreatment with BAY11-7082 significantly attenuated the histopathologic changes of burn-induced ALI. We also measured the protein concentrations in BALF (Figure 4(c)) and MPO activity (Figure 4(d)) in lung tissues and observed that pretreatment with BAY11-7082 significantly decreased the protein concentrations and MPO activity. Both the IL-1*β* and IL-18 levels in lung tissues were measured via qRT-PCR. The levels of IL-1*β* and IL-18 (Figures 4(e) and 4(f)) were significantly decreased in lungs pretreated with BAY11-7082.

### 3.5. BAY11-7082 Inhibited NLRP3 Inflammasome Signaling in AMs Challenged with Burn Serum and Attenuated Both IL-1*β* and IL-18 Expression

**Figure 5:**
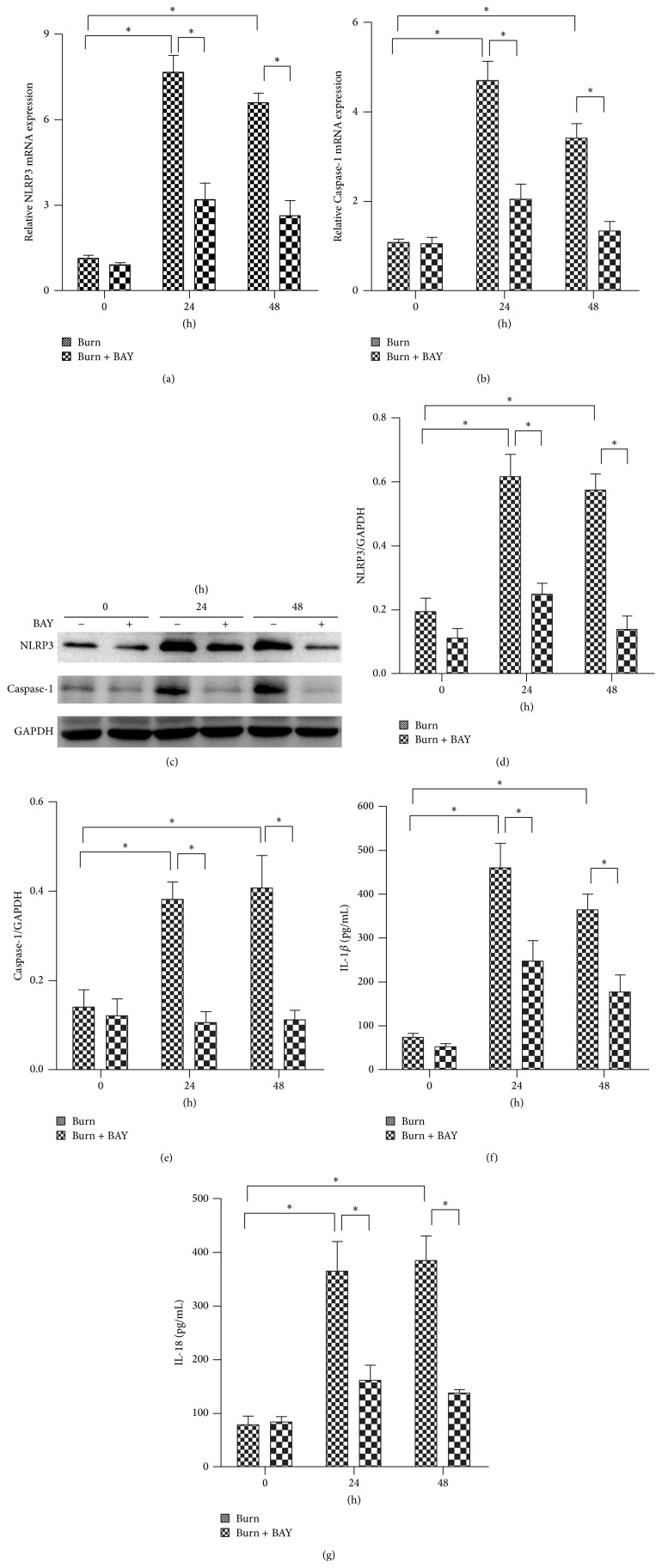
BAY11-7082 inhibited the NLRP3 inflammasome signals of AMs challenged with burn serum. AMs cells were exposed to 10% sham serum (0 h in burn group), 10% sham serum + BAY11-7082 (0 h in burn + BAY group), 10% burn serum, and 10% burn serum + BAY11-7082 for 24 h or 48 h. BAY11-7082 was treated immediately after serum challenge. Real-time PCR showed the mRNA expression ((a) and (b)) of NLRP3 and Caspase-1 in macrophages exposed to burn serum for 24 h or 48 h. Immunoblots showed the protein level change ((c), (d), and (e)) of NLRP3 and Caspase-1 in each group. The release of IL-1*β* (f) and IL-18 (g) in the supernatant was tested by ELISA. Results represent mean ± SEM of six independent experiments. ^*∗*^ *P* < 0.05. AM, alveolar macrophage.

To mimic a burn injury microenvironment, AMs were exposed to either 10% sham serum or 10% burn serum collected from rats subjected to 30% TBSA burns, either with or without BAY11-7082. As demonstrated in Figures 5(a), 5(b), 5(c), 5(d), and 5(e), the mRNA and protein levels of NLRP3 and Caspase-1 were significantly elevated 24 h and 48 h after the burn serum challenge. When treated with BAY11-7082, both the mRNA and protein levels of NLRP3 and Caspase-1 were significantly decreased. The release of IL-1*β* (Figure 5(f)) and IL-18 (Figure 5(g)) in AMs supernatant followed a similar trend.

### 3.6. A ROS Scavenger Inhibited NLRP3 Inflammasome Activity in AMs Exposed to Burn Serum

**Figure 6:**
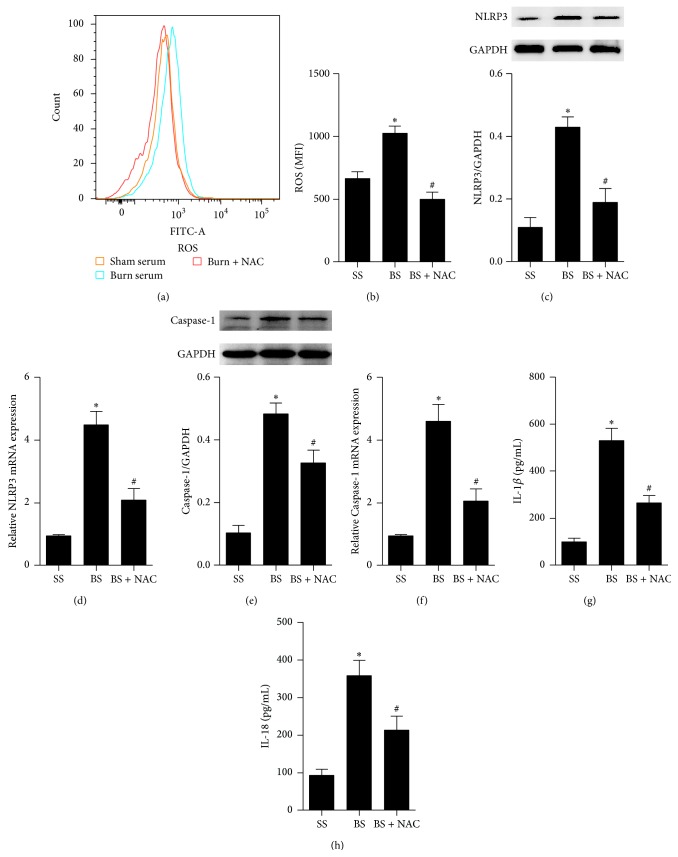
ROS scavenger inhibited the activation of NLRP3 inflammasome in AMs exposed to burn serum. AMs were challenged with burn serum with NAC, a ROS scavenger, for 24 h or 48 h. ROS were examined by way of FACS (a) and were quantified by way of mean fluorescence intensity (b). The activation of NLRP3 ((c) and (d)) and Caspase-1 ((e) and (f)) was measured by immunoblots or real-time PCR. The release of IL-1*β* (g) and IL-18 (h) in the supernatant was tested by ELISA. Results represent mean ± SEM of six independent experiments. ^*∗*^ *P* < 0.05 compared to the value at SS group. ^#^ *P* < 0.05 compared to the value at BS group. SS, sham serum; BS, burn serum; AM, alveolar macrophage.

ROS play a key role in controlling inflammasome activity triggered by ischemia reperfusion injury. To determine whether the NLRP3 inflammasome activity depended on ROS production, AMs were treated with NAC, a ROS scavenger, and exposed to burn serum. ROS production significantly increased in AMs exposed to burn serum (Figures 6(a) and 6(b)). NAC significantly decreased the NLRP3 and Caspase-1 activity (Figures 6(c), 6(d), 6(e), and 6(f)). Additionally, NAC also decreased the release of IL-1*β* and IL-18 in cell supernatants (Figures 6(g) and 6(h)).

## 4. Discussion

ALI is responsible for significant morbidity and mortality among patients suffering from extensive burn injuries [32]. The lung is continuously exposed to a variety of inhaled infectious agents and host-derived danger signals. Therefore, the lung is frequently the first organ to fail, even in the absence of inhalational injury [33]. In the present study, we successfully established a 30% TBSA full-thickness burn-induced ALI rat model, similar to those utilized in previous studies, based on observed lung histologic changes, inflammatory mediator infiltration, and pulmonary microvasculature permeability.

Increasing amounts of evidences have demonstrated that inflammatory mediators and cytokines induce the migration of activated inflammatory cells into the air spaces; the mediators released by these inflammatory cells result in tissue damage associated with ALI and ARDS [9]. Blocking excessive inflammation may modify these systemic responses and decrease the amount of tissue damage sustained in either ALI or ARDS. Macrophages are pivotal in orchestrating the immune response and may have multiple functions that can upregulate or downregulate host defense mechanisms [34]. Activated macrophages express numerous receptors linked to ALI and can both produce and release numerous substances that play essential roles in induction and regulation of acquired immune and inflammatory reactions [35]. Alveolar macrophages comprise 90% of cells found in the BALF under inflammatory conditions [10], which indicates that alveolar macrophages play a central role in the development of ALI.

Burn injury is a complex pathophysiological process; following a severe burn, hypovolemic shock develops, and a rapid increase of cytokines and chemokines in inflammatory cascade is initiated [1]. Therapies that target leukocytes or inflammatory mediators significantly reduce burn injury [36]. IL-1*β* activates additional inflammatory cells and releases more inflammatory mediators and cytokines, which amplifies the injury signals and triggers an inflammatory cascade. IL-18 also functions as a proinflammatory cytokine and serves as an autocrine activator that facilitates the expression of other inflammatory mediators. IL-1*β* was elevated in serum and lung homogenates within the first few hours following burn injury in both humans and rodents. IL-18 was also significantly increased in lymphoid cell supernatants and in lung and intestinal tissue homogenates obtained from rats subjected to 25% TBSA burns in combination with increased lung and intestinal MPO levels and edema [16, 37]. The inhibition of IL-1*β* or IL-18 attenuated ALI severity following different damage [7, 14]. Our data demonstrated that IL-1*β* and IL-18 concentrations in serum and lung tissue homogenates gradually increased within 12 h and peaked either 24 h or 48 h after injury.

IL-1*β* and IL-18 are translated in the form of precursors. The expression of these proinflammatory cytokines is regulated by two checkpoints: (1) transcription and (2) maturation as well as release from the cell. Previous studies have demonstrated that Caspase-1, also known as IL-1*β*-converting enzyme, converts pro-IL-1*β* and pro-IL-18 into their biologically active forms. Therefore, Caspase-1 is also a key mediator in the formation of inflammatory cascade. In fact, the activation of Caspase-1 facilitates both cleavage and maturation of a variety of protein precursors, many of which ultimately play a role in the cytoskeleton, glycolysis [38], mitochondria function [39], and inflammation. Recently, Osuka et al. [40] observed Caspase-1 activity in both macrophages and dendritic cells 4 h after burn injury, and this activity peaked within 1 day; Caspase-1 activity secondary to burn injury was also noted in NK cells, CD4 T cells, and B cells. Rana et al. [16] observed that rats treated with AC-YVAD-CHO (a Caspase-1 inhibitor) exhibited decreased MPO activity and tissue edema 24 h after alcohol-induced burn injury. Our data indicated that both the mRNA and protein levels of Caspase-1 were significantly enhanced following burn injury, whereas, when the upstream molecule NLRP3 was inhibited, the expression of Caspase-1 was also reduced with ALI alleviated.

Mature IL-1*β* and IL-18 are produced when Caspase-1 cleaves inactive pro-IL-1*β* and pro-IL-18 precursors. This cleavage is activated by a large multiprotein complex known as inflammasomes. Of these inflammasomes, the NLRP3 inflammasome is most extensively studied. The primary function of NLRP3 inflammasome is to upregulate and activate the innate immune system via secretion of IL-1*β* and IL-18. Therefore, we speculated that the NLRP3 inflammasome plays an important role in ALI following burn injury. In fact, Stanojcic et al. [41] and Diao et al. [42] observed activation of the NLRP3 in white adipose tissue of burn patients; however, the mechanism controlling NLRP3 inflammasome activity after burn insult is still not elucidated and the function of NLRP3 in ALI following burn injury is not well understood. We observed that both the protein and mRNA levels of NLRP3 gradually increased in a time-dependent manner. The upregulation of NLRP3 can be attributed to two reasons. One is migration and aggregation of inflammatory cells into lungs and the other is activity of NLRP3 inflammasome signaling. So separating leukocytes from lung epithelia and separately assessing inflammasome component expression in these compartments are essential for further research. BAY11-7082 administration inhibited NLRP3 and significantly attenuated ALI, which indicated that NLRP3 activity following burn injury was detrimental to the lungs and that inhibition of NLRP3 inflammasome signaling protects tissues in the setting of burn-induced ALI. However, the mechanism by which burn injury induces inflammasome activity and IL-1*β* and IL-18 maturation remains unclear.

Various intracellular events caused by cellular stress, including alterations in mitochondrial regulation, lysosomal stability, and ion concentrations, can activate the NLRP3 inflammasome after their accumulation under conditions of tissue damage [43]. The generation of ROS is one of the most important factors in activation of NLRP3. Previous studies have demonstrated that myocardial mitochondrial ROS levels were significantly elevated in a 40% TBSA burn injury model [44]. Furthermore, ROS-induced oxidative stress mediated the development of burn-induced ALI [45]. Therefore, we speculated that burn injury activates the inflammasome via production of ROS.

Earlier in the paper, we elaborated the critical role played by macrophages in ALI. As AMs comprise 90% of cells found in the BALF under inflammatory conditions, AMs were utilized for* in vitro *experiments designed to study NLRP3 expression and the signaling pathways that trigger the activation of the NLRP3 inflammasome in burn-induced ALI. To mimic the burn injury microenvironment, we challenged AMs with 10% (vol/vol) serum collected from rats subjected to 30% TBSA burns. The NLRP3 inflammasome inhibitor BAY11-7082 and the ROS scavenger NAC were used in our study.

Our results indicated that burn serum strongly increased the expression levels of NLRP3, Caspase-1, IL-1*β*, and IL-18. These levels significantly decreased following BAY11-7082 treatment. To study the relationship between NLRP3 activity and ROS, we analyzed the NLRP3 activity in the presence of ROS scavenger. The results indicated that both the ROS and NLRP3 activities were significantly increased in burn serum-challenged group following stimulation. NAC administration significantly decreased the NLRP3 activity. Therefore, NLRP3 inflammasome signaling is likely a partial ROS-dependent process. However, the precise mechanism by which ROS modulate the NLRP3 activity warrants further investigation. As NAC is a broad-spectrum ROS scavenger, it can eliminate not only the mitochondria-derived ROS but also the cytoplasm-derived ROS. To clearly distinguish which part mainly regulated NLRP3 inflammasome, further study is needed. In this experiment, we used AMs and RAW 264.7 cells (data not shown) to study the mechanism controlling NLRP3 activation. However, in the procedure of pulmonary acute inflammatory response, alveolar macrophages were activated firstly, and then bone-marrow-derived macrophages immigrated and aggregated to the pulmonary mesenchyme. Therefore, study in bone-marrow-derived macrophages is also essential. These all will be the focus of our future studies.

**Figure 7:**
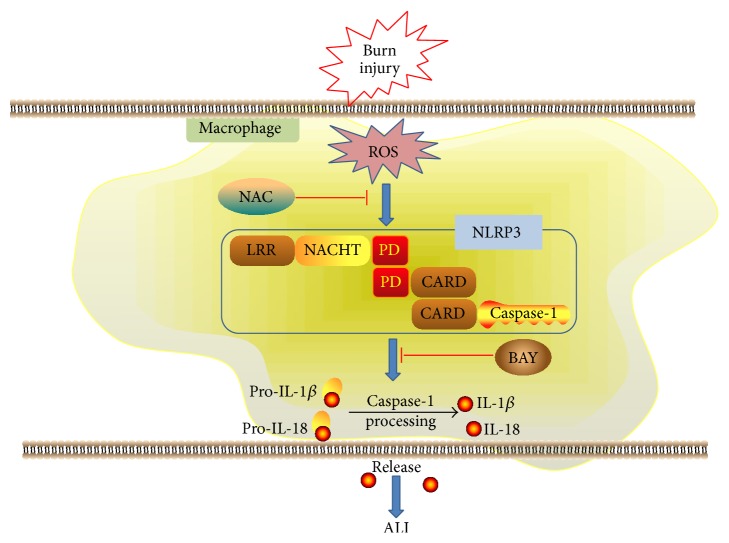
Model of ROS-mediated NLRP3 inflammasome activation in burn-induced acute lung injury. Burn injury activates ROS in macrophages and in turn the elevated ROS promote the activation of NLRP3 inflammasome and subsequent IL-1*β* and IL-18 secretion. IL-1*β* and IL-18 act as inflammation initiating cytokines and magnify inflammatory response. The amplified inflammatory responses lead to ALI. Blocking NLRP3 or ROS activation can alleviate ALI.

This study demonstrated that burn injury activates the NLRP3 inflammasome in AMs, which promotes the release of inflammatory initiating cytokines, such as IL-1*β* and IL-18, and amplifies the inflammatory response to cause ALI. Additionally, burn injury-induced NLRP3 inflammasome activity is a partial ROS-dependent process. Inhibition of either NLRP3 or ROS activity alleviates ALI (Figure 7). These results suggest that modulation of either NLRP3 or ROS activity may represent a promising therapeutic strategy in the setting of severe burn injury.

## Figures and Tables

**Table 1:** Names and sequences of primers used for quantitative RT-PCR.

Name	Species	Forward primer	Reverse primer
NLRP3	Rat	CAGCGATCAACAGGCGAGAC	AGAGATATCCCAGCAAACCTATCCA
Caspase-1	Rat	ACTCGTACACGTCTTGCCCTCA	CTGGGCAGGCAGCAAATTC
IL-1*β*	Rat	CCCTGAACTCAACTGTGAAATAGCA	CCCAAGTCAAGGGCTTGGAA
IL-18	Rat	GACTGGCTGTGACCCTATCTGTGA	TTGTGTCCTGGCACACGTTTC
GAPDH	Rat	GAACATCATCCCTGCATCCA	CCAGTGAGCTTCCCGTTCA
